# Machine learning−derived multivariable predictors of postcardiotomy cardiogenic shock in high-risk cardiac surgery patients

**DOI:** 10.1016/j.xjon.2024.10.002

**Published:** 2024-10-09

**Authors:** Edward G. Soltesz, Randi J. Parks, Elise M. Jortberg, Eugene H. Blackstone

**Affiliations:** a Department of Thoracic and Cardiovascular Surgery, Cleveland Clinic, Cleveland, Ohio; b Academic Research, Abiomed, Danvers, Mass

**Keywords:** CABG, mitral valve surgery, LV dysfunction, machine learning, postcardiotomy shock, STS ACSD

## Abstract

**Objective:**

To develop a model for preoperatively predicting postcardiotomy cardiogenic shock (PCCS) in patients with poor left ventricular (LV) function undergoing cardiac surgery.

**Methods:**

From the Society of Thoracic Surgeons Adult Cardiac Database, 11,493 patients with LV ejection fraction ≤35% underwent isolated on-pump surgery from 2018 through 2019, of whom 3428 experienced PCCS. In total, 68 preoperative clinical variables were considered in machine-learning algorithms trained and optimized using scikit-learn software.

**Results:**

Compared with patients with ideal recovery, those that did were younger (65 vs 67 years), more likely female, Black, with low LV ejection fraction (26.5 vs 28.9%), previous myocardial infarction, chronic lung disease, diabetes, reoperation, or advanced heart failure. Among those with PCCS versus ideal recovery, operative mortality was 27% (925/3428) versus 0.1% (5/8065). PCCS occurred more often after coronary artery bypass grafting with concomitant mitral valve repair or after longer perfusion and clamp times. Reliable preoperative PCCS predictors were more advanced cardiac, liver, and renal failure; frailty; and greater white cell count. Out of sample test set receiver operating curve achieved an area under the curve of 0.74 with acceptable calibration Hosmer-Lemeshow statistic χ^2^ = 1.33, *P* = .25.

**Conclusions:**

In patients with severe LV dysfunction undergoing cardiac surgery, risk of PCCS is elevated by preoperative failure of other organ systems and complexity of the planned operation that prolongs myocardial ischemia and cardiopulmonary bypass. This risk calculator could serve as an important tool to preoperatively identify patients in need of advanced levels of support.

## Graphical abstract

Postcardiotomy cardiogenic shock risk model performance, risk predictors, and risk stratification.

**Figure:**
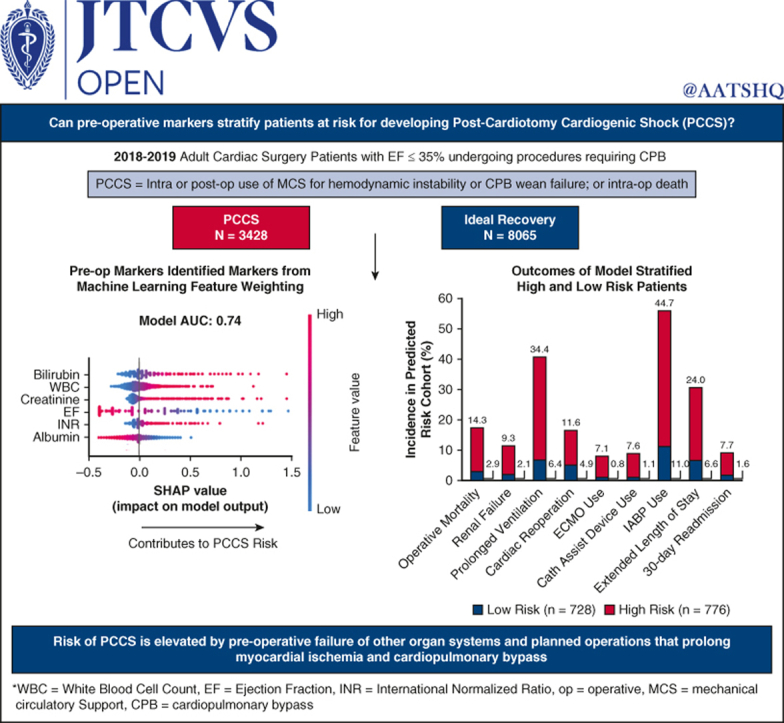

**Figure:**
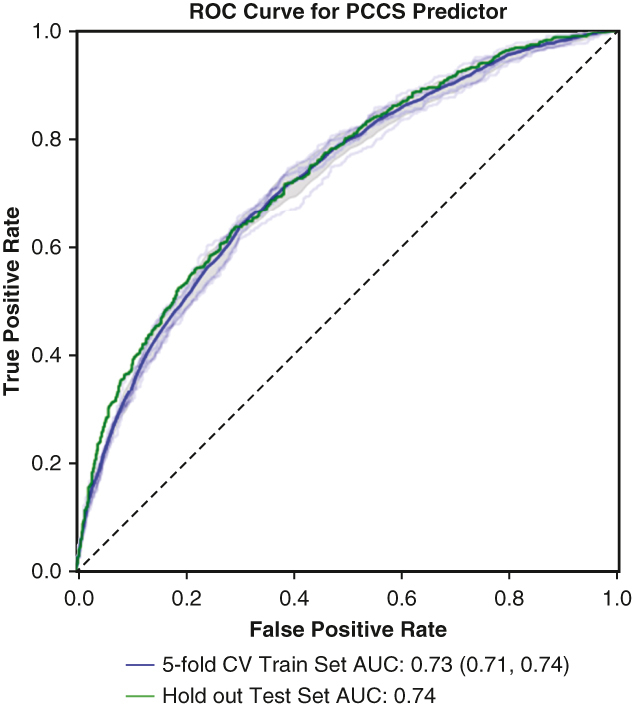
ROC curve for machine-learning derived PCCS risk score.

**Central Message**

A machine learning−derived model was developed that accurately predicts PCCS in patients with poor LV function. Major risk factors included preoperative failure of other organ systems and complexity of the planned operation.

**Perspective**

Patients with left ventricular dysfunction undergoing cardiac surgery present a therapeutic challenge. Although such patients with treatable cardiac lesions typically have the most to gain, their risk of PCCS remains high. Development of a risk calculator could serve as an important tool to preoperatively identify patients in need of advanced levels of support.

Risk of mortality after cardiac surgery is dramatically elevated in the subset of patients who develop postcardiotomy cardiogenic shock (PCCS).^1^ PCCS has been cited as occurring in roughly 2% to 6% of all adults undergoing cardiac surgery and carries a mortality of 50% to 80%.^2^, ^3^, ^4^ Clinically characterized by a cardiac index <2.2 L/min/m^2^, hypotension (systolic blood pressure <90 mm Hg or the need for vasoactive medications to maintain systolic blood pressure >90 mm Hg) unresponsive to volume resuscitation, and signs of impaired end-organ perfusion, PCCS is typically manifest as an inability to wean from cardiopulmonary bypass (CPB) in the operating room or rapid deterioration of cardiac function in the early postoperative period. Smedira and Blackstone^5^ found younger age, number of reoperations, emergency operation, greater levels of creatinine, greater left ventricular dysfunction, and history of myocardial infarction significant predictors of PCCS. More recently, Javorski and colleagues^6^ found that the occurrence of PCCS was very high among patients with low ejection fraction (EF); 28% among patients with ischemic cardiomyopathy (ICM) and 27% among patients without ICM and was predicted by right heart dysfunction among patients with ICM and greater cardiac decompensation in patients without ICM.

Accurate prediction of PCCS is critical in order to appropriately identify patients preoperatively who might benefit from strategies that may mitigate the consequences of prolonged end-organ insult such as preemptive temporary mechanical circulatory support, preoperative adjudication for advanced therapies, or transfer to a tertiary high-volume center facile in caring for such high-risk phenotypes.^7^, ^8^, ^9^ Our objective was therefore to develop and validate a machine-learning model for preoperatively predicting PCCS in patients with poor left ventricular (LV) function undergoing cardiac surgery using readily available national registry-level data.

## Methods

### Definitions

PCCS requiring mechanical circulatory support (MCS) or life-saving measure (herein PCCS) was defined as either (1) intra- or postoperative use of MCS for hemodynamic instability or failure to wean from CPB (Appendix E1 Supplemental Definitions) or (2) intraoperative cardiac death. Ideal recovery was defined as having all of the following: (1) no mechanical circulatory support usage, and (2) no prolonged ventilation, and (3) no delayed sternal closure, and (4) no postoperative end-organ dysfunction, (5) time in intensive care unit <10 days, (6) discharged alive, and (7) no readmission.

### Data Source

A fully deidentified analytic dataset was furnished by the Society of Thoracic Surgeons (STS) Research Center subsequent to a data request from industry that was processed and approved by STS. Analysis of these deidentified data sets has been approved as research exempt from Cleveland Clinic Institutional Review Board review.

### Patient Selection

**Figure 1:**
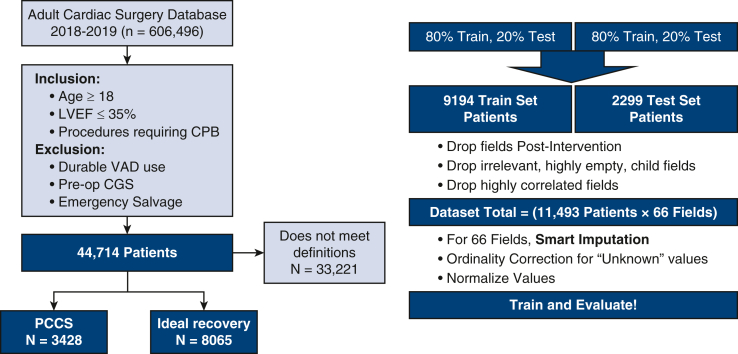
Attrition chart of patient assignment to PCCS and ideal recovery cohorts. *LVEF*, Left ventricular ejection fraction; *CPB*, cardiopulmonary bypass; *VAD*, ventricular assist device; *CGS*, cardiogenic shock; *PCCS*, postcardiotomy cardiogenic shock.

The STS Adult Cardiac Surgery Database was queried from 2018 to 2019 for patients 18 years or older with LV EF ≤35% who underwent isolated on-pump surgery. Patients with a durable left ventricular assist device, preoperative cardiogenic shock, or emergency salvage status were excluded, yielding 44,714 operations (Figure 1).

Of the 44,714 interventions that met our baseline inclusion and exclusion criteria (Figure 1), we then applied the PCCS and ideal recovery labels per the cohort definitions described previously to assign each group. In total, 3428 (29.8%) patient interventions met the PCCS cohort requirements and 8065 (70.2%) patient interventions met the ideal recovery cohort requirements. The remaining 33,221 (74.3%) interventions did not meet the requirements for either cohort and consequentially were not included in this analysis.

### End Points

Thirty-day and in-hospital postoperative outcomes were assessed per STS definitions. Postoperative length of stay and 30-day readmission were assessed in patients surviving to discharge.

### Machine Learning

We identified 195 relevant variables captured before surgery and up to start of intervention. All measurements were those taken closest to the date/time of surgery but before anesthetic management. Variables with a percentage of missing values >30% were excluded and correlated variables were collapsed to a minimum set. Race, ethnicity, and administrative variables were not considered. Empty categorical variables were imputed as the “not documented” value where available, else were imputed by random selection from the population-level variable distribution. Missing values for continuous variables were imputed with the median. The values were then corrected for ordinality such that severity increased with assigned value (ie, an unknown value in the STS database assigned as 5 was reassigned to −1 and variables such as TobaccoUse assigned such that frequency or severity increased from 0: none to 5: current). Each operation was represented by its major components (coronary artery bypass grafting [CABG], aortic valve replacement, mitral valve replacement, mitral valve repair, and other). Two derived features were added: (1) pr_count was constructed from the variables to estimate the number of components that made up the operation, as this would be estimated during surgical planning and (2) an estimated table time, TableTm, which was a multiplicative of the number of diseased vessels and surgery type. A final set of 68 variables was considered.

The 11,493 operations were randomly split 80%/20% into Training (n = 9194) and Hold-out Testing (n = 2299) datasets for modeling. Multiple models were considered in the analysis including: logistic regression, support vector classification, decision tree, random forest, Gaussian Naive Bayes, and XGBoost. Hyperparameters were optimized using Grid Search. Feature selection was performed using recursive feature estimation with cross validation (CV) with the estimator matching the model architecture per model training. Sixty-two features were identified with this method. Modeling was performed using scikit-learn^10^ and XGBoost^11^ Python software. We then trained models with selected number of inputs on the basis of feature importance weights, ranging from 5 to all variables in order of 62 variable coefficients, aiming to minimize the number of features required for a simpler predictor.

### Model Evaluation

For each training iteration, the receiver operator curve (ROC) was calculated, which compares true and false positives rates. The area under the curve (AUC) statistic [0-1] was used to evaluate each model and their relative predictive power. The estimate range during model training of the ROC-AUC was evaluated by using 5-fold CV within the training dataset by which random subsets were assigned to fit the model versus evaluate the ROC-AUC. We report the average across the folds as well as the [minimum, maximum] AUC. Because of the imbalanced nature of the dataset, we also evaluated the precision recall curve, which contextualizes the tradeoff between precision and recall for PCCS predictions. We compare the precision recall curve-AUC to the prevalence of PCCS in the training patient set.

To create a model with reduced variables, we subselected a set of variables from the previous training exercise with high model coefficients. These variables were added back in one at a time and average AUC reported. Once the AUC stopped improving per variable added, we selected the model with variables up to this iteration.

We calculated the SHapley Additive exPlanations (SHAP) values to examine each variable’s impact on a given prediction. Positive SHAP values indicate the variable pushes the model towards the positive class, whereas negative pushes the model to the negative class. The absolute value of SHAP values indicates the relative importance of a given variable to the output.

### Statistical Analysis

Demographics, medical history, surgical procedure details, and clinical outcomes were summarized for patients who developed PCCS and those with ideal recovery. Clinical outcomes were also summarized for patients based on their PCCS risk assessment.

All calculated *P* values are 2-sided. Statistical analysis was performed using SAS statistical software version, 9.4 (SAS Institute).

## Results

### Patient Baseline Characteristics

**Figure 2:**
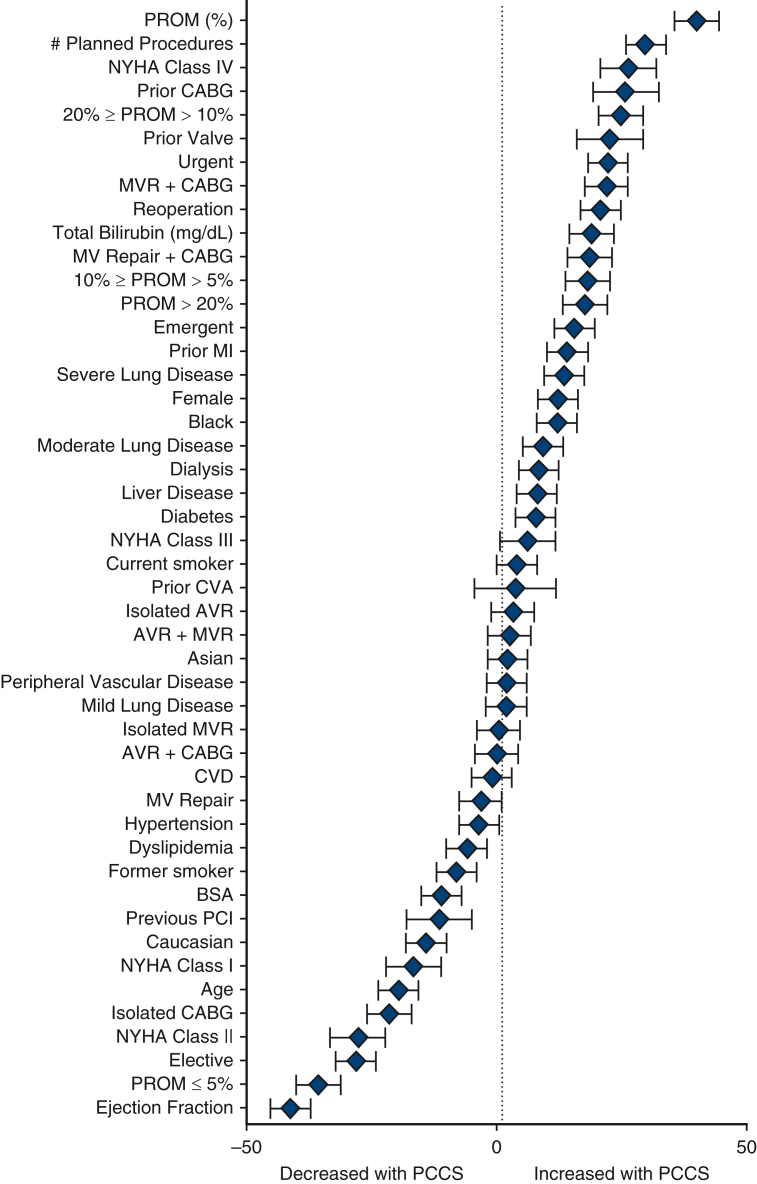
Absolute standardized difference plot of baseline characteristics between PCCS and ideal recovery cohorts derived from Table E1. Shown are standardized differences in percentage (%) difference (with 95% confidence interval bars) of a variable PCCS-ideal recovery. *PROM*, Predicted risk of mortality; *NYHA*, New York Heart Association; *CABG*, coronary artery bypass grafting; *MVR*, mitral valve replacement; *MV*, mitral valve; *MI*, myocardial infarction; *CVA*, cerebrovascular accident; *AVR*, aortic valve replacement; *CVD*, cerebrovascular disease; *BSA*, body surface area; *PCI*, percutaneous coronary intervention; *PCCS*, postcardiotomy cardiogenic shock.

Compared with patients who had ideal recovery, those who had PCCS were younger (65.2 ± 10.50 vs 67.2 ± 9.58 years) and more likely to be female (24.8% vs 19.7%) (Table E1). The factors most highly correlated with increased occurrence of PCCS include the number of planned procedures, New York Heart Association Class IV, previous CABG or valve, predicted risk of mortality, urgent operations, reoperation, and total bilirubin (Figure 2). Baseline characteristics most negatively correlated with PCCS included greater EF, elective operations, New York Heart Association Class II, and older age.

### Clinical Outcomes With PCCS

**Figure 3:**
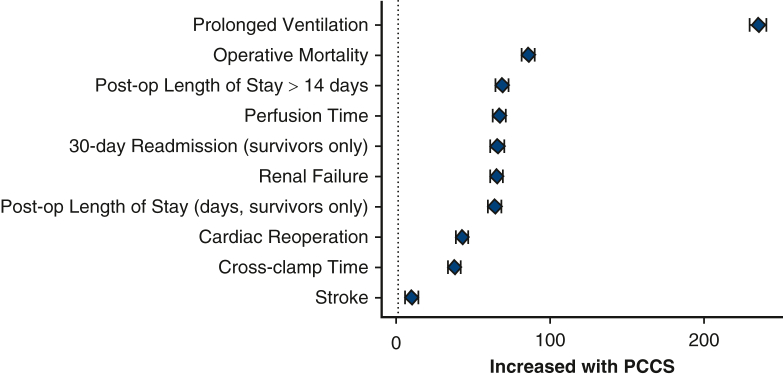
Absolute standardized difference plot of outcomes between PCCS and ideal recovery cohorts derived from Table E2. Shown are standardized differences in percentage (%) difference (with 95% confidence interval bars) of a variable PCCS-ideal recovery. *PCCS*, Postcardiotomy cardiogenic shock.

Occurrence of all reported adverse outcomes was greater in the PCCS cohort (Figure 3, Table E2). The greatest difference was in occurrence of prolonged ventilation (73.4% vs 0%; *P* < .0001), followed by operative mortality (27.0% vs 0.1%; *P* < .0001). Postoperative length of stay, 30-day readmission, renal failure, cardiac reoperation, and stroke also occurred more often in the PCCS cohort. They were more likely to experience longer perfusion times (155 ± 80.1 minutes vs 111 ± 46.1 minutes; *P* < .0001) and crossclamp times (99 ± 50.4 minutes vs 82.6 ± 38.0 minutes; *P* < .0001).

### Machine-Learning Model

The training dataset consisted of 2742 (30%) patients with PCCS and 6452 (70%) with ideal recovery patients. The testing dataset consisted of 686 (30%) patients with PCCS and 1613 (70%) with ideal recovery.

**Figure 4:**
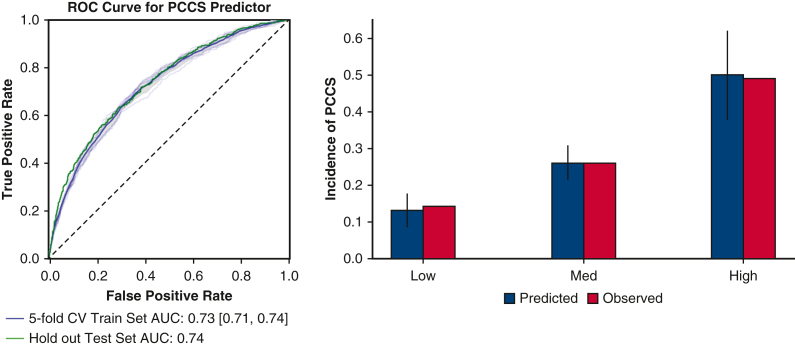
*Right*, ROC of PCCS prediction across (*blue*) 5-fold cross validation training dataset (n = 9194) and (*green*) out of sample test patient set (n = 2299). *Left*, Calibration of the PCCS risk model. Predicted risk quantiles versus observed PCCS percentages of test set patients (acceptable calibration, Hosmer-Lemeshow χ^2^ = 1.33, *P* = .25). *ROC*, Receiver operating curve; *PCCS*, postcardiotomy cardiogenic shock; *CV*, cross-validation; *AUC*, area under the curve.

**Figure 5:**
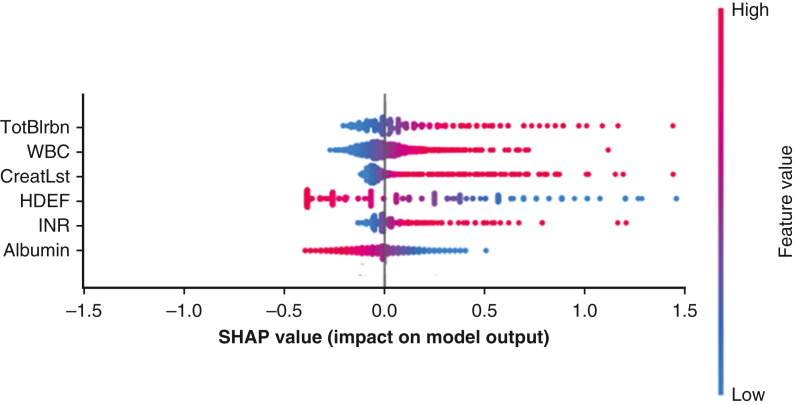
SHAP value (log-odds space) of highly weighted feature coefficients from fitted multivariate logistic regression model. Variable SHAP values >0 indicate an increase on model output (greater PCCS risk) where color indicates the variable range (ie, high levels of bilirubin [TotBlrbn] are associated with a greater PCCS risk). *WBC*, White blood cell; *CreatLst*, last creatinine level; *HDEF*, hemodynamic data- ejection fraction; *INR*, international normalized ratio; *SHAP*, SHapley Additive exPlanations; *PCCS*, postcardiotomy cardiogenic shock.

The models had similar AUC-ROC values across 5-fold CV, between 0.70 and 0.73. The authors chose to perform final model evaluation using logistic regression because of its inherent linearity although we recognize these other model architectures are viable. The logistic regression model trained on 62 variables gave a 5-fold CV across training set AUC-ROC of 0.73 [0.71, 0.74] and a test set AUC-ROC of 0.74 (Figure 4). The precision-recall curve had an AUC of 0.56 where the percentage of PCCS samples in the training data was 30%. The predicted values were separated into 3 equal quantiles for low-medium-high categorization to compare with the observed probability and achieved an acceptable calibration with Hosmer-Lemeshow statistic of χ^2^ = 1.33, *P* = .25. Figure 5 illustrates the impact on the model output for a subset of the top feature coefficients using SHAP values.^12^

**Figure 6:**
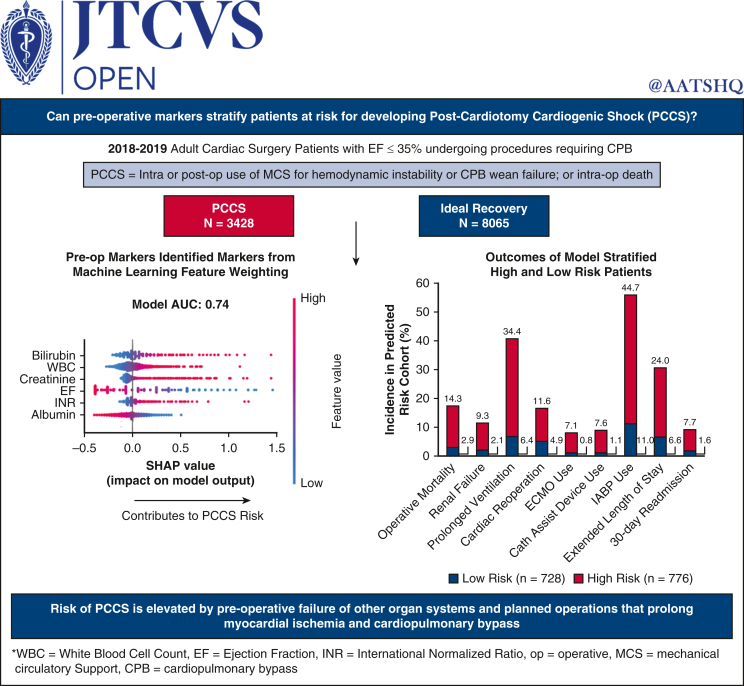
Pre-op marker stratification highlights impact of advanced end organ failure, frailty and elevated WBC on PCCS development risk. The model designated high and low risk groups experienced a significant difference in outcomes post-operatively. *PCCS*, Post-cardiotomy cardiogenic shock; *EF*, ejection fraction; *CPB*, cardiopulmonary bypass; *Op*, operative; *MCS*, mechanical circulatory support; *WBC*, white blood cell count; *INR*, International normalized ratio; *ECMO*, extracorporeal membrane oxygenation; *IABP*, intra aortic baloon pump.

The 3 quantiles generated by the PCCS risk model demonstrated statistical significance between the low- and high-risk groups in the majority of surgical outcomes assessed (Table 1). With the exception of stroke, operative mortality, renal failure, prolonged ventilation, cardiac reoperation, postoperative length of stay, and 30-day readmission were all significantly greater in the high-risk group compared with the low-risk group. Use of extracorporeal membrane oxygenation (ECMO), catheter-based assist device, and intra-aortic balloon pump were all significantly greater in the high- versus low-risk group (Figure 6).

**Table 1:** Surgical outcomes among the 3 quantiles generated by the full PCCS risk model

Parameter	Low risk (n = 728)	Medium risk (n = 795)	High risk (n = 776)	*P* value (low vs high)
Operative mortality	2.9% (21/728)	7.3% (58/795)	14.3% (111/776)	<.0001
Renal failure	2.1% (15/728)	5.0% (40/795)	9.3% (72/776)	<.0001
Prolonged ventilation	6.4% (45/707)	15.4% (115/748)	34.4% (242/704)	<.0001
Stroke	3.4% (24/707)	3.9% (29/747)	3.1% (22/702)	.7830
Cardiac reoperation, % (n/N)	4.9% (36/728)	6.5% (52/795)	11.6% (90/776)	<.0001
ECMO use	0.8% (6/728)	2.8% (22/795)	7.1% (55/776)	<.0001
Catheter-based assist device use	1.1% (8/728)	2.1% (17/795)	7.6% (59/774)	<.0001
IABP use	11.0% (80/728)	23.5% (187/795)	44.7% (347/776)	<.0001
VAD use	0.3% (2/728)	1.0% (8/795)	0.9% (7/773)	.1801
Postoperative length of stay >14 d, % (n/N)	6.6% (48/727)	11.6% (92/794)	24.0% (186/774)	<.0001
Postoperative length of stay, d	707	748	677	<.0001
Mean (SD)	8.0 (4.40)	9.5 (5.78)	13.1 (12.64)	
Median (Q1-Q3)	7.0 (6.0-9.0)	8.0 (6.0-11.0)	9.0 (7.0-14.0)	
Min-max	1-41	0-55	0-120	
30-d readmission, % (n/N)	1.6% (11/704)	2.9% (21/736)	7.7% (51/662)	<.0001

*ECMO*, Extracorporeal membrane oxygenation; *IABP*, intra-aortic balloon pump; *VAD*, ventricular assist device; *SD*, standard deviation; *Q1*, first quartile; *Q3*, third quartile; *PCCS*, postcardiotomy cardiogenic shock.

We identified a training set AUC-ROC plateau at N = 12 features, achieving a 5-fold CV of 0.71 [0.69, 0.72], a test set AUC-ROC of 0.71, and acceptable calibration of χ^2^ = 2.96, *P* = .09. The precision-recall curve had an AUC of 0.51. The top feature coefficients were bilirubin, international normalized ratio, white blood cell count, albumin, EF, creatinine, surgical component count, age, previous CABG, status, TableTm, and other surgery type.

The quantiles generated by the N = 12 PCCS risk model show significantly worse clinical outcomes in the high-risk group in comparison with the low-risk group (Table E3). With the exception of stroke, operative mortality, renal failure, prolonged ventilation, cardiac reoperation, postoperative length of stay, and 30-day readmission were all significantly more common in the high versus the low-risk group. Use of ECMO, catheter-based assist device, and intra-aortic balloon pump were all significantly greater in the high- versus low-risk group.

## Discussion

In this study, we developed a novel, explainable machine-learning model to reliably predict the development of PCCS from common preoperative registry variables in patients with LV dysfunction undergoing cardiac surgery. To the authors’ knowledge, this study used the largest, multicenter dataset in training a predictive risk model to stratify patient risk of PCCS that can be applied before surgery. Because patients with LV dysfunction are at greatest risk for PCCS, it is critically important to fully characterize their perioperative risk profile before planned surgical intervention so as to allow appropriate triage to centers capable of high-risk surgery as well as to inform whether the patient is even a candidate for conventional cardiac surgery or should be referred for advanced therapies (left ventricular assist device or transplant). Furthermore, characterizing the risk of PCCS preoperatively allows for earlier institution of temporary MCS as an adjunct to the planned cardiac surgery. Temporary MCS promotes myocardial perfusion and recovery through LV unloading and normalizing cardiac output. Earlier institution of temporary MCS could theoretically reduce or eliminate the toxic effects of high-dose inotropes and vasopressors, the deleterious effects of prolonged low cardiac output, or excessive periods of CPB during which temporary MCS is deployed. In addition, accurate PCCS risk assessment would prevent costly device overuse in all patients with LV dysfunction. We believe that PCCS risk prediction must be made preoperatively to have the greatest clinical benefit, unlike the machine-learning algorithm developed by Morisson and colleagues that predicts the need for veno-arterial (VA)-ECMO after the development of low cardiac outcome syndrome/PCCS.^13^

In developing the PCCS risk model, we aimed to include variables commonly available in electronic health record (EHR) systems such that the model could prospectively evaluate risk from the existing patient chart. As such our PCCS risk logistic regression model, trained on 62 variables, achieved an AUC-ROC of 0.74. This compliments Morisson’s VA-ECMO Implantation for patients with post-cardiotomy-low cardiac outcome syndrome predictive score. While Morisson examined the specific need for VA-ECMO treatment with intra- and postsurgery factors and post-PCCS development, our model considered only presurgical factors such that individuals at high-risk of PCCS may be preemptively addressed to prevent the need of reactive treatment.^12^ Embedding our risk model in EHR allows surgical risk considerations, such as PCCS, to be readily available when evaluating and triaging patients for cardiac surgery. Although trained on a multisite dataset, data drift and model generalization remain a concern for machine-learning derived risk scores. The EHR-embedded approach for clinical risk models such as ours enables model tuning and validation in a given hospital system to evaluate clinical utility. Readily available online risk calculators, such as the STS predicted risk of mortality, are widely used for providing clinical decision support. The reduced-input analysis of the PCCS risk model showed that the 12 identified variables provide significant PCCS risk stratification to be similarly deployed in an online setting where variables are manually entered. Finally, the simple PCCS clinical risk score discriminated risk quantiles well with eleven data elements. Such a score can be useful for bedside clinicians for rapid risk assessment.

Currently, the PCCS risk model captures preoperative variables as a surgical planning tool. However, the risk profile of developing PCCS changes as one observes patient response peri- and postoperatively. By embedding the risk model into an EHR, the model could theoretically update temporally in response to changing patient status throughout admission. A fully connected hospital data system expands machine learning enabled clinical decision support capability to consider real-time patient data in addition to EHR. In future work, we anticipate augmenting the modeling to include high-resolution waveforms, such as electrocardiogram and hemodynamic monitoring data, for continuous, real-time point-of-care PCCS risk assessment.

This study was designed to evaluate the performance of a machine learning−based predictive algorithm on external data. Further refining of the model can easily be accomplished with increased granular data and a narrower clinical definition of PCCS. The model could theoretically also include preoperative hemodynamic data, viability assessment, and more granular catheterization data.

There are several limitations to this study. As with all registries, the STS Adult Cardiac Surgery Database collects a set number of data fields. For example, some information on the severity of cardiogenic shock, such as lactate level and hemodynamics, are not captured. Perioperative low cardiac output syndrome or PCCS is not captured, and thus a study-specific definition for PCCS was created that is conservative because of its reliance on device usage and, therefore, undoubtedly underestimates the true incidence as most patients are treated solely with inotropes and vasopressors. Furthermore, the choice of MCS device might not necessarily reflect a clinical need but rather institutional availability and/or surgeon preference. In addition, details as to the specific device, timing, and indications for MCS escalation of care at the patient level are not available.

Because of the rarity of PCCS development and severity of the mortality outcomes, our aim for this first-of-its-kind risk assessment was to catch as many high-risk patients as possible with significant probability of detection such that protective action may be considered. In training a machine-learning classifier, the objective is to discriminate between features in the n-dimensional feature space such that datapoints assigned to each binary class is separable. Given the data source structure, with PCCS indicators captured in MCS usage fields, we estimated conservatively to get reflective test statistics on model performance. Evaluating the model excluded patients yielded a 12-point increase on patients who experienced cardiac death within hospital (non-operating room) versus patients who did not die. Although we cannot make clinical conclusions on this limited information, the trend stood whereby patients who died with all-cause cardiac mortality demonstrated increased risk score.

In patients with severe LV dysfunction undergoing cardiac surgery, risk of PCCS is elevated by preoperative failure of other organ systems and complexity of the planned operation that prolongs myocardial ischemia and cardiopulmonary bypass. This novel, explainable machine-learning model reliably predicts the development of PCCS from common preoperative registry variables and can serve as an important tool to preoperatively identify patients in need of advanced levels of support.

### Webcast

You can watch a Webcast of this AATS meeting presentation by going to: https://www.aats.org/resources/machine-learning-derived-risk-7368.

**Figure:**
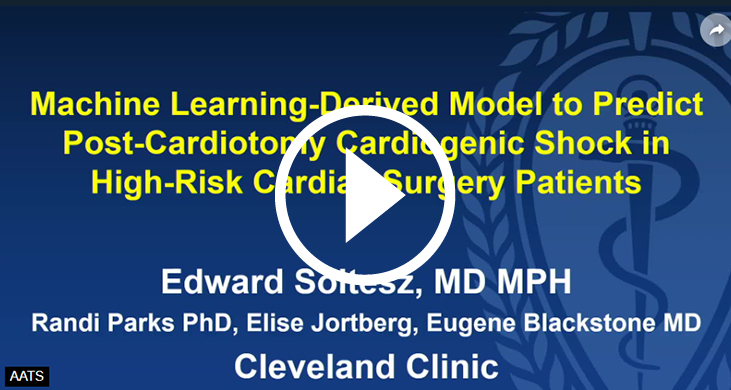

## Conflict of Interest Statement

E.G.S. reports honoraria from Abiomed, AtriCure, Abbott, and Edwards. R.J.P. and E.M.J. are employees of Abiomed. E.H. B. reports honoraria from Abiomed.

The *Journal* policy requires editors and reviewers to disclose conflicts of interest and to decline handling or reviewing manuscripts for which they may have a conflict of interest. The editors and reviewers of this article have no conflicts of interest.

## Figures and Tables

**Table E1:** Baseline characteristics of the PCCS and ideal recovery cohorts

Parameter	PCCS (n = 3428)	Ideal recovery (n = 8065)	Standardized difference (% [95% CI] PCCS − No PCCS)	*P* value
Age	3428	8065	−19.6 (−23.6, −15.6)	<.0001
Mean (SD)	65.2 (10.50)	67.2 (9.58)		
Median (Q1-Q3)	66.0 (58.0-73.0)	68.0 (61.0-74.0)		
Min-max	24-90	20-96		
Gender, % (n/N)				
Female	24.8% (851/3427)	19.7% (1590/8063)	12.3 (8.3-16.3)	<.0001
Male	75.2% (2576/3427)	80.3% (6473/8063)	−12.3 (−16.3, −8.3)	<.0001
Race, % (n/N)				
Asian	3.3% (114/3428)	3.0% (238/8065)	2.1 (−1.8, 6.1)	.2864
Black	11.5% (393/3428)	7.9% (636/8065)	12.1 (8.1-16.1)	<.0001
White	75.7% (2595/3428)	81.5% (6569/8065)	−14.1 (−18.1, −10.0)	<.0001
Other	5.1% (176/3428)	3.9% (315/8065)	5.9 (1.9-9.9)	.0029
Multiple	1.3% (46/3428)	1.2% (96/8065)		
Not reported	3.0% (104/3428)	2.6% (211/8065)		
BSA, n	3419	8055	−11.0 (−15.0, −7.0)	<.0001
Mean (SD)	1.985 (0.2430)	2.012 (0.2366)		
Median (Q1-Q3)	1.982 (1.815-2.143)	2.009 (1.854-2.167)		
Min-max	1.18-2.88	1.24-2.99		
Dialysis, % (n/N)	5.8% (200/3420)	4.0% (323/8059)	8.5 (4.5-12.5)	<.0001
Dyslipidemia, % (n/N)	84.5% (2886/3416)	86.6% (6972/8055)	−5.9 (−9.9, −1.9)	.0035
Hypertension, % (n/N)	88.2% (3014/3417)	89.3% (7193/8053)	−3.5 (−7.5, 0.5)	.0811
Diabetes, % (n/N)	55.8% (1910/3423)	51.9% (4182/8057)	7.8 (3.8-11.8)	.0001
Ejection fraction, n	3428	8065	−41.2 (−45.2, −37.1)	<.0001
Mean (SD)	26.5 (6.34)	28.9 (5.57)		
Median (Q1-Q3)	27.5 (23.0-32.0)	30.0 (25.0-33.0)		
Min-max	5-35	1-35		
Liver disease, % (n/N)	5.1% (174/3407)	3.5% (279/8035)	8.1 (4.1-12.1)	<.0001
Chronic lung disease, % (n/N)				
No	56.4% (1886/3342)	63.7% (5043/7922)	−14.8 (−18.8, −10.7)	<.0001
Mild	14.3% (477/3342)	13.6% (1078/7922)	1.9 (−2.1, 6.0)	.3498
Moderate	9.3% (312/3342)	6.8% (539/7922)	9.3 (5.3-13.4)	<.0001
Severe	12.1% (406/3342)	8.1% (639/7922)	13.6 (9.5-17.6)	<.0001
Severity unknown	7.8% (261/3342)	7.9% (623/7922)	−0.2 (−4.2, 3.8)	.9218
CVD, % (n/N)	24.1% (818/3393)	24.5% (1969/8039)	−0.9 (−4.9, 3.1)	.6617
Previous CVA, % (n/N)	43.4% (353/814)	41.5% (813/1959)	3.8 (−4.4, 11.9)	.3649
Peripheral vascular disease, % (n/N)	16.4% (560/3417)	15.7% (1262/8057)	2.0 (−2.0, 6.0)	.3310
Status, % (n/N)				
Elective	26.0% (892/3427)	39.0% (3147/8061)	−28.0 (−32.1, −24.0)	<.0001
Emergent	3.9% (132/3427)	1.4% (111/8061)	15.6 (11.6-19.6)	<.0001
Urgent	70.1% (2403/3427)	59.6% (4803/8061)	22.2 (18.2-26.2)	<.0001
Total bilirubin, mg/dL, n	3114	7209	19.1 (14.8-23.3)	<.0001
Mean (SD)	0.81 (0.728)	0.69 (0.440)		
Median (Q1-Q3)	0.70 (0.50-1.00)	0.60 (0.40-0.80)		
Min-max	0.1-26.4	0.1-11.0		
Previous myocardial infarction, % (n/N)	66.4% (2250/3388)	59.6% (4781/8021)	14.1 (10.1-18.2)	<.0001
NYHA class, % (n/N)				
Class I	2.7% (52/1951)	6.1% (224/3700)	−16.7 (−22.1, −11.2)	<.0001
Class II	16.0% (313/1951)	27.4% (1012/3700)	−27.7 (−33.2, −22.2)	<.0001
Class III	48.5% (947/1951)	45.5% (1682/3700)	6.2 (0.7-11.7)	.0273
Class IV	32.8% (639/1951)	21.1% (782/3700)	26.4 (20.9-31.9)	<.0001
PROM, % (n/N)				
PROM >20%	2.6% (73/2797)	0.4% (32/7197)	17.7 (13.4-22.1)	<.0001
20% ≥ PROM > 10%	10.2% (284/2797)	3.8% (277/7197)	24.9 (20.5-29.3)	<.0001
10% ≥ PROM > 5%	20.2% (564/2797)	13.4% (963/7197)	18.2 (13.9-22.6)	<.0001
PROM ≤5%	67.1% (1876/2797)	82.3% (5925/7197)	−35.6 (−40.0, −31.2)	<.0001
PROM, %	2797	7197	40.0 (35.6-44.4)	<.0001
Mean (SD)	5.119 (5.5708)	3.291 (3.2746)		
Median (Q1-Q3)	3.280 (1.800-6.270)	2.240 (1.310-4.060)		
Min-max	0.32-73.84	0.21-40.14		
Reoperation, % (n/N)	7.8% (268/3422)	3.1% (252/8055)	20.8 (16.8-24.8)	<.0001
Active tobacco use, % (n/N)				
Current smoker	25.6% (872/3407)	23.8% (1918/8043)	4.1 (0.0-8.1)	.0464
Former smoker	38.4% (1307/3407)	42.3% (3400/8043)	−8.0 (−12.0, −4.0)	.0001
Never smoker	36.0% (1228/3407)	33.9% (2725/8043)	4.5 (0.5-8.5)	.0260
Previous percutaneous coronary intervention, % (n/N)	71.7% (933/1301)	76.7% (2016/2628)	−11.4 (−18.1, −4.8)	.0007
Previous CABG, % (n/N)	13.4% (174/1296)	5.9% (154/2627)	25.8 (19.2-32.5)	<.0001
Previous valve, % (n/N)	11.5% (149/1297)	5.3% (138/2627)	22.7 (16.0-29.3)	<.0001
Total number of planned procedures	3428	8065	29.8 (25.8-33.9)	<.0001
Mean (SD)	2.0 (1.28)	1.7 (1.07)		
Median (Q1-Q3)	2.0 (1.0-3.0)	1.0 (1.0-2.0)		
Min-max	0-8	0-7		

*PCCS*, Postcardiotomy cardiogenic shock; *CI*, confidence interval; *SD*, standard deviation; *Q1*, first quartile; *Q3*, third quartile; *BSA*, body surface area; *CVD*, cerebrovascular disease; *CVA*, cerebrovascular accident; *NYHA*, New York Heart Association; *PROM*, predicted risk of mortality; *CABG*, coronary artery bypass grafting.

**Table E2:** Procedures and outcomes of the PCCS and ideal recovery cohorts

Parameter	PCCS (n = 3428)	Ideal recovery (n = 8065)	Standardized difference (% [95% CI] PCCS-No PCCS)	*P* value
Surgery, % (n/N)				
Isolated CABG	68.4% (1967/2876)	77.8% (5757/7396)	−21.4 (−25.7, −17.1)	<.0001
Isolated AVR	0.1% (4/2876)	0.0% (3/7396)	3.3 (−1.0, 7.6)	.1015
Isolated MVR	3.2% (92/2876)	3.1% (232/7396)	0.4 (−4.0, 4.7)	.8716
AVR + CABG	8.4% (242/2876)	8.4% (620/7396)	0.1 (−4.2, 4.4)	.9587
MVR + CABG	7.3% (211/2876)	2.6% (193/7396)	21.9 (17.6-26.2)	<.0001
AVR + MVR	1.2% (35/2876)	0.9% (70/7396)	2.6 (−1.7, 6.9)	.2210
MV repair	2.0% (57/2876)	2.4% (180/7396)	−3.1 (−7.4, 1.2)	.1708
MV repair + CABG	9.3% (268/2876)	4.6% (341/7396)	18.6 (14.3-22.9)	<.0001
Perfusion time	3420	8055	66.8 (62.7-70.9)	<.0001
Mean (SD)	154.5 (80.06)	110.9 (46.10)		
Median (Q1-Q3)	136.0 (101.0-187.0)	102.0 (78.0-134.0)		
Min-max	22-722	1-449		
Crossclamp time	3223	7635	37.6 (33.5-41.8)	<.0001
Mean (SD)	99.4 (50.43)	82.6 (37.97)		
Median (Q1-Q3)	89.0 (63.0-124.0)	75.0 (55.0-102.0)		
Min-max	0-394	3-316		
Operative mortality	27.0% (925/3428)	0.1% (5/8065)	85.6 (81.5-89.8)	<.0001
Renal failure	17.5% (599/3428)	0% (0/8065)	65.1 (61.0-69.2)	<.0001
Prolonged ventilation	73.4% (1995/2717)	0% (0/8065)	235.1 (229.7-240.4)	<.0001
Stroke	4.5% (123/2713)	2.7% (218/8062)	9.8 (5.5-14.2)	<.0001
Cardiac reoperation	16.5% (564/3428)	3.8% (310/8065)	42.7 (38.7-46.7)	<.0001
Postoperative length of stay >14 d	31.8% (1087/3415)	6.3% (511/8063)	68.6 (64.5, 72.7)	<.0001
Postoperative length of stay, d	2621	8063	64.0 (59.5-68.5)	<.0001
Mean (SD)	15.1 (14.39)	8.3 (4.30)		
Median (Q1-Q3)	11.0 (7.0-18.0)	7.0 (6.0-10.0)		
Min-max	0-372	0-139		
30-d readmission	17.7% (441/2490)	0% (0/8065)	65.6 (61.0-70.2)	<.0001

*PCCS*, Postcardiotomy cardiogenic shock; *CI*, confidence interval; *CABG*, coronary artery bypass grafting; *AVR*, aortic valve replacement; *MVR*, mitral valve replacement; *MV*, mitral valve; *SD*, standard deviation; *Q1*, first quartile; *Q3*, third quartile.

**Table E3:** Surgical outcomes among the 3 quantiles generated by the 12-feature PCCS risk model

Parameter	Low risk (n = 519)	Medium risk (n = 1035)	High risk (n = 745)	*P* value (low vs high)
Operative mortality	3.5% (18/519)	7.3% (76/1035)	12.9% (96/745)	<.0001
Renal failure	2.3% (12/519)	4.9% (51/1035)	8.6% (64/745)	<.0001
Prolonged ventilation	7.0% (35/499)	15.6% (155/991)	31.7% (212/669)	<.0001
Stroke	4.2% (21/499)	3.6% (36/989)	2.7% (18/668)	.1546
Cardiac reoperation	6.4% (33/519)	7.0% (72/1035)	9.8% (73/745)	.0299
ECMO use	1.0% (5/519)	2.5% (26/1035)	7.0% (52/745)	<.0001
Catheter-based assist device use	0.8% (4/519)	2.7% (28/1035)	7.0% (52/743)	<.0001
IABP use	11.9% (62/519)	22.3% (231/1035)	43.1% (321/745)	<.0001
LV assist device use	0.2% (1/519)	0.8% (8/1035)	1.1% (8/742)	.0902
Postoperative length of stay >14 d	7.1% (37/519)	11.6% (120/1033)	22.7% (169/743)	<.0001
Postoperative length of stay, d	504	968	660	<.0001
Mean (SD)	8.2 (4.80)	9.6 (7.54)	12.4 (11.29)	
Median (Q1-Q3)	7.0 (6.0-9.0)	8.0 (6.0-11.0)	9.0 (7.0-13.0)	
Min-max	0-39	0-116	1-120	
30-d readmission	1.2% (6/501)	3.6% (34/957)	6.7% (43/644)	<.0001

*ECMO*, Extracorporeal membrane oxygenation; *IABP*, intra-aortic balloon pump; *LV*, left ventricular; *SD*, standard deviation; *Q1*, first quartile; *Q3*, third quartile; *PCCS*, postcardiotomy cardiogenic shock.

## Appendix E1 Supplemental Definitions

“Failure to wean from cardiopulmonary bypass” was described per mechanical circulatory support type. “Failure to wean from cardiopulmonary bypass” was an indication in the “Primary Use” reason for mechanical circulatory support (intra-aortic balloon pump, catheter-based assist devices, and ventricular assist device) data fields in the Society of Thoracic Surgeons Adult Cardiac Surgery Database. For venoarterial extracorporeal membrane oxygenation, the specific selection from field values were as follows: implanted intraoperative or implanted postoperative with the primary reason being cardiac failure. Failing to wean as defined in the data specification requires a “failure to separate from the heart-lung machine.”
